# Research Engagement With Persons Living With Dementia: Putting Lessons Learned Into Practice

**DOI:** 10.1093/geroni/igaa057.2585

**Published:** 2020-12-16

**Authors:** Lori Frank

**Affiliations:** RAND Corporation, Arlington, Virginia, United States

## Abstract

In the US few research initiatives actively engage persons living with dementia (PLWD) as partners in the research. The 2017 Summit actively engaged multiple types of stakeholder groups, including one for Persons Living with Dementia (PLWD), and was the first large-scale US research meeting to actively engage PLWD in planning and conduct of the meeting. The PLWD conducted a self-evaluation of their work that yielded best practices, meeting the need for guidelines for engaging with PLWD. The 2020 Summit presented the opportunity to test best practices. Some were implemented by the group conveners, like use of video-enabled meetings. Others were implemented with the PLWD, including decisions about governance structure for the group. The use of learnings from the first Summit in engaging with PLWD in the following Summit supported refinement of some engagement practices, yielding a list of recommendations for future work.

